# Country-level structural stigma, identity concealment, and day-to-day discrimination as determinants of transgender people’s life satisfaction

**DOI:** 10.1007/s00127-021-02036-6

**Published:** 2021-02-13

**Authors:** Richard Bränström, John E. Pachankis

**Affiliations:** 1 Division of Psychology, Department of Clinical Neuroscience, Karolinska Institutet, Nobels väg 9, 171 77 Stockholm, Sweden; 2 Department of Social and Behavioral Sciences, Yale School of Public Health, New Haven, CT USA

**Keywords:** Transgender, Concealment, Minority stress, Life-satisfaction, Structural stigma, LGBT

## Abstract

**Purpose:**

Discriminatory laws, policies, and population attitudes, surrounding transgender people vary greatly across countries, from equal protection under the law and full acceptance to lack of legal recognition and open bias. The consequences of this substantial between-country variation on transgender people’s health and well-being is poorly understood. We therefore examined the association between structural stigma and transgender people’s life satisfaction across 28 countries.

**Methods:**

Data from transgender participants (*n* = 6771) in the 2012 EU-LGBT-survey regarding identity concealment, day-to-day discrimination, and life satisfaction were assessed. Structural stigma was measured using publicly available data regarding each country’s discriminatory laws, policies, and population attitudes towards transgender people.

**Results:**

Multilevel models showed that country-level structural stigma was associated with lower life satisfaction, an association largely explained by higher levels of identity concealment in higher-structural-stigma countries. Yet identity concealment was also associated with lower day-to-day discrimination and therefore protected against even lower life satisfaction.

**Conclusion:**

The results emphasize the importance of changing discriminatory legislation and negative population attitudes to improve transgender people’s life satisfaction, and also highlight targets for intervention at interpersonal and individual levels.

**Supplementary Information:**

The online version contains supplementary material available at 10.1007/s00127-021-02036-6.

## Introduction

Studies increasingly show that transgender people, namely those who experience incongruity between their sex assigned at birth and current gender identity, are at particular risk of mental health concerns, psychological distress, and other indicators of poor life satisfaction [1, 2]. In particular, studies have reported a higher incidence of psychiatric problems, such as anxiety and depression [3, 4], and a higher degree of suicidal ideation and suicide attempts among transgender people compared to the general population [3, 5].

Transgender people’s higher risk of poor mental health is believed to be a consequence of their increased exposure to stigma and minority stress compared to individuals without transgender experience, that is, cis-gender individuals [6, 7]. Stigma occurs and can influence the mental health and wellbeing of transgender people at multiple levels. At the structural level, stigma is expressed as unjust laws, policies, and cultural norms that deny, or fail to protect, the equal rights of minority populations [5, 8, 9]. At the interpersonal level, stigma manifests as discrimination in social interactions, victimization, non-affirmation of gender identity, and other expressions of prejudice in interactions between individuals [7, 10, 11]. At the individual level, stigma can reduce transgender people’s coping resources by requiring maladaptive behavioral strategies, such as concealment of transgender identity and social isolation [12], as well as internalized forms of transphobia and negative expectations for future events [7]. The extent to which transgender people are exposed to these types of interpersonal and individual-level experiences is believed to be a consequence of stigma at a structural level and the degree of transgender hostility in the environment surrounding a transgender individual [8, 9]. Each of these forms of stigma is believed to put transgender people at increased risk of poor mental health [6].

Structural stigma is usually measured as discriminatory laws and policies and prejudicial attitudes towards the stigmatized [9]. Examining the relationship between structural stigma and the health of the stigmatized requires large datasets with sufficient numbers of stigmatized respondents distributed across geographical units, such as countries, that are diverse in terms of structural stigma. Because most previous studies of transgender health and life experiences have been conducted in small samples within a single country or municipality, larger-scale examinations of the association between structural stigma on transgender people’s well-being have not previously been possible [1, 6]. One exception is a study conducted in the US that found that structural stigma at the state level was associated with lower odds of lifetime suicide attempts among transgender adults [5]. Yet, in this study, structural stigma toward sexual minority individuals (e.g., those who identify as gay and lesbian) was used as a proxy for transgender-specific structural stigma due to the insufficient variation in transgender-specific structural stigma across US states at the time of study. Research has since identified the importance of using structural stigma indicators specific to the population of study [13]. Because transgender people have distinct health determinants from sexual minority individuals [6], such as legal facilitators of and barriers to gender affirmation [2], examining the association between transgender-specific structural stigma and health-related outcomes represents an important research aim not previously addressed. Because structural stigma surrounding transgender people is currently highly variable across European countries, European-wide studies of transgender people represent a suitable opportunity for examining associations between transgender-specific structural stigma and indicators of this population’s health and wellbeing.

**Fig. 1:**
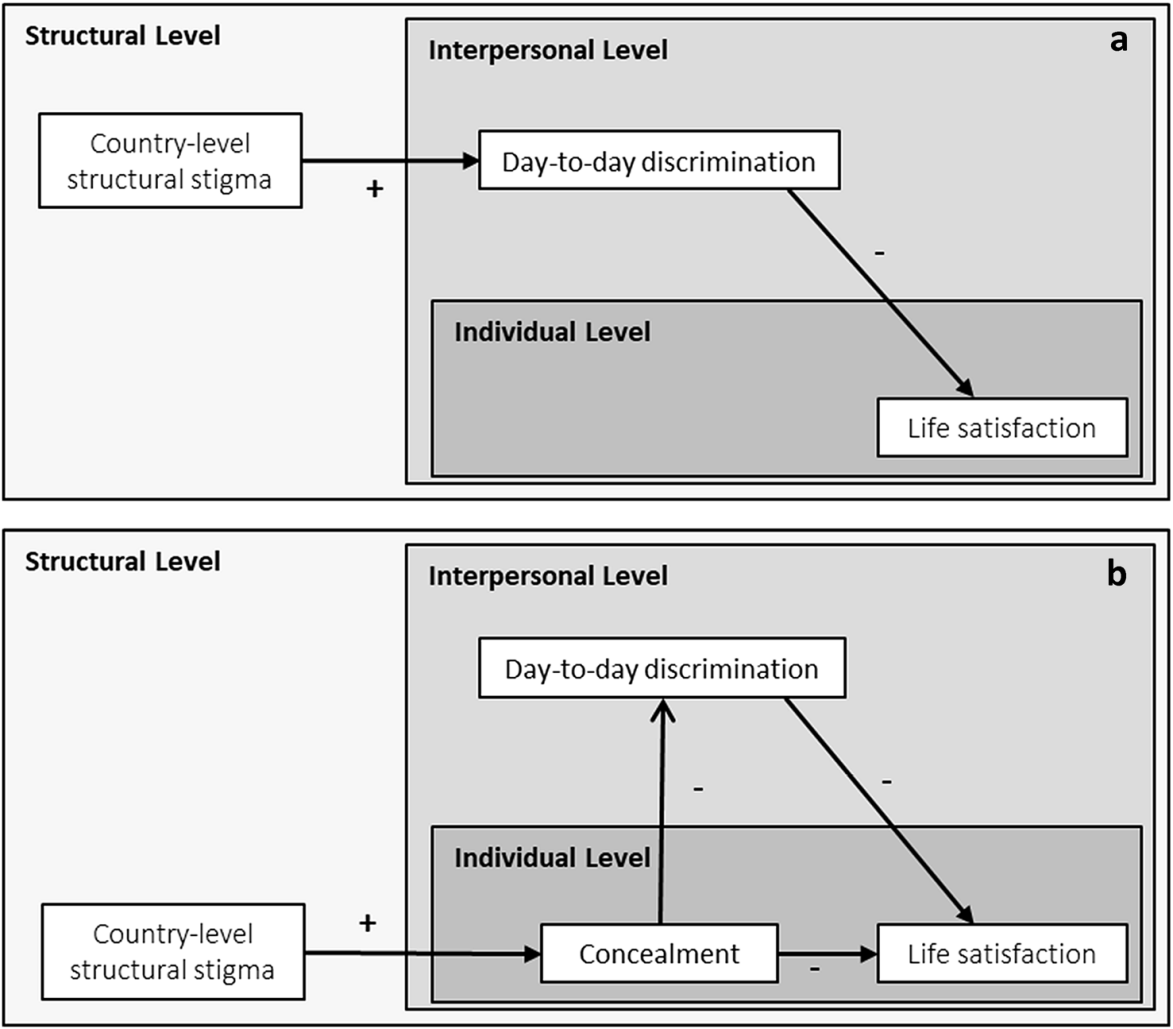
**a**, **b** Conceptual model of associations among structural, interpersonal, and individual levels of stigma predicting life-satisfaction among transgender people

The purpose of this study was to investigate the association between transgender-specific structural stigma, measured as country-level laws, policies, and community attitudes toward transgender people, and transgender people’s life satisfaction. We further explored the role of interpersonal- and individual-level forms of stigma in explaining this association. We specifically hypothesized that the association between structural stigma and life satisfaction would be mediated by exposure to discrimination, an interpersonal form of stigma consistently associated with poor health-related outcomes [14]. According to this hypothesis, we expected that transgender people would be exposed to more day-to-day discrimination in countries with greater structural stigma, which compromises their life satisfaction (see Fig. 1a). We also hypothesized that the association between structural stigma and life satisfaction would be mediated through transgender identity concealment. Following this hypothesis, we expected that transgender people would be more likely to conceal their gender identity in countries with greater structural stigma, thereby explaining their lower life satisfaction in those countries. At the same time, because concealment can protect against discrimination [15], we also hypothesized a serial mediation whereby structural stigma predicts greater concealment to predict lower discrimination to predict life satisfaction. In this case, the association between structural stigma and poor life satisfaction is expected to be smaller than it otherwise would be if discrimination and its association with lower concealment were not taken into account (see Fig. 1b).

To explore these hypotheses, we took advantage of the European Union Lesbian, Gay, Bisexual, and Transgender (EU-LGBT) survey, containing one of the largest-known international samples of transgender people. In addition to covering a geographic area diverse in structural stigma surrounding transgender people, the EU-LGBT survey contains a validated measure of life satisfaction that has demonstrated a strong link with other important indicators of health and wellbeing [16, 17], in addition to measures of interpersonal (i.e., discrimination) and individual (e.g., identity concealment) variables that hypothetically operate as mechanisms between structural stigma and life satisfaction. To our knowledge, this is the first international investigation of structural stigma and transgender people’s wellbeing. It is also the first study to examine potential individual and interpersonal mechanisms that could explain this association.

## Methods

### Study sample

This study uses data from the European Union Lesbian, Gay, Bisexual, and Transgender (EU-LGBT) survey [18], which surveyed the treatment of and wellbeing of lesbian, gay, bisexual, and transgender (LGBT) individuals in 28 European countries using a web-based survey between April and July 2012. The sample included individuals 18 years of age or older who identified as LGBT and lived in one of the 28-member states of the European Union. Participants were recruited via internet advertisements posted on over 400 local, national, and international LGBT websites and via national LGBT organizations. The survey was completed by a total of 93,079 participants, 6771 (7.3%) of whom self-identified as transgender. The survey development and methods have been described in detail elsewhere [18].

### Self-report measures

#### Transgender identity

Individuals were classified based on their responses to an item assessing current or life-time experiences as a transgender individual, with the question: “Are you/have you been a transgender person?” with response options: “yes,” or “no.” A total of 6771 individuals self-identified as transgender in the survey.

#### Life-satisfaction

Participants’ general life satisfaction was assessed with the question: “All things considered, how satisfied would you say you are with your life these days?” Respondents indicated their response on a scale from 1 (very dissatisfied) to 10 (very satisfied). This question was the same as a question used in the World Values Survey [19], in which it has been asked every year from 1981 to 2009 and shown high consistency (*r* = 0.78) across time and countries [17]; it has also shown high stability within individuals [20] and has consistently been shown to be linked to numerous indicators of health and wellbeing [16, 17].

#### Concealment of transgender identity

Concealment of transgender identity was assessed as degree of openness about transgender identity to the following groups of people: “family members,” “friends,” “neighbors,” and “work colleagues/schoolmates,” with the question: “To how many people among the following groups are you open about yourself being transgender?” Response options included the following: “none [coded = 0],” “a few [coded = 1],” “most [coded = 2],” “all [coded = 3].” We calculated an average score based on the openness indicated across the four groups above. Concealment was dichotomized at an average score across the four groups of 1.0 (i.e., “a few”), so that participants with an average score of less than 1.0 were categorized as ‘concealed’ and those scoring 1.0 or higher as ‘not concealed.’

#### Everyday discrimination

An adapted eight-item scale was used to assess respondents’ exposure to everyday discrimination [21]. In response to the question: “In the last 6 months, in your day-to-day life, how often have any of the following things happened to you because you are or are assumed to be transgender?,” respondents were asked to indicate exposure to events, such as “You have been treated with less courtesy than other people,” and “You have been treated with less respect than other people.” Response options included the following: “never happened in the last sixth months [coded = 0],” “happened only once in the last 6 months [coded = 1],” “2–5 times in the last 6 months [coded = 3.5],” and “6 times or more in the last 6 months [coded = 6].” Responses to the eight items were summed as an indicator of perceived discrimination during the past 6 months.

#### Covariates

Age, gender, relationship status, ethnic minority status, education, annual household income, type of living area (i.e., ‘urban’ or ‘rural’), and country of birth were included as individual-level socio-demographic factors.

### County-level characteristics

#### Country-level structural stigma

We created a measure of structural stigma related to transgender identity in 2012 for all EU countries following similar prior research on structural stigma toward other stigmatized populations [22]. We first created an index of laws and policies concerning transgender people collected by the International Lesbian, Gay, Bisexual, Trans and Intersex Association in Europe [23]. The index of laws and policies was created by summing across four domains of discrimination and protections (i.e., asylum provisions based on gender identity, constitutional and legal protections against discrimination toward transpeople, marriage recognition for transgender people, legal gender recognition). Each form of discrimination was assigned a negative point, whereas each form of protection was assigned a positive point. These points were then summed to create a scale from − 7 to 13. We then combined this index with a measure of social attitudes from the European Commission’s survey: “Special Eurobarometer 393—Discrimination in the EU in 2012,” a European population-based survey of 26,622 participants’ responses regarding attitudes towards social issues [24]. To assess attitudes towards transgender people, participants were asked to rate to what degree they would feel comfortable with a transgender person in the highest political position in their country: “Using a scale from 1 to 10, please tell me how you would feel about having a transgender person in the highest elected political position in [COUNTRY]? Where ‘1’ means that you would feel “totally uncomfortable” and “10” that you would feel “totally comfortable”.” The index of laws and policies and the measure of population attitudes were both standardized by *z*-transformation and then averaged to create one score of structural stigma for each country. The correlation between the two measures was 0.31. The final structural stigma score varied between the lowest score in the United Kingdom − 2.12 and the highest score in Latvia 1.56, with a higher value indicating a higher degree of structural stigma.

#### Gini coefficient

To adjust for each country’s income equality—a known correlate of life satisfaction and positive attitudes towards sexual minorities—the Gini coefficient in 2012 for each country was included as a country-level covariate [25]. The Gini coefficient varies between 0 and 1, whereby a low value indicates equality and a high value indicates major inequalities.

#### County-level life-satisfaction

Because the association between country-level structural stigma toward transgender persons and life satisfaction could potentially be explained by general country-level differences in life satisfaction, we controlled for average country-level self-reported life satisfaction as assessed in the general population of each country using the population-based European Social Survey [26].

### Statistical analysis

To analyze data at both individual and country levels, multi-level models were used. Individual-level factors (i.e., life satisfaction, concealment, discrimination, and socio-demographic factors) were modeled at level 1 and country-level factors (i.e., structural stigma, Gini coefficient, and population-average life-satisfaction) were modeled at level 2. Fixed effects were estimated using maximum likelihood parameter estimation. The amount of missing data varied from 0% for sociodemographic variables (e.g., gender assigned at birth, age, country of residence) to 0.9% for life satisfaction. Only respondents with complete records on all study variables were included in analyses. The analyses were conducted using MPlus statistical software and the level for statistical significance in regression models was set at *α* = 0.05.

First, three separate models were calculated to estimate the association between country-level structural stigma and the primary outcome (i.e., life satisfaction) and the two proposed mediating variables (i.e., concealment of transgender identity and day-to-day discrimination). Second, since the association between structural stigma and concealment was significant, a multi-level mediation analysis was performed to estimate the indirect effect of country-level structural stigma and life satisfaction through concealment. Since the association between structural stigma and discrimination was non-significant, we did not examine an indirect effect of country-level stigma on life-satisfaction via discrimination. Third, to determine if concealment might protect sexual minorities from day-to-day discrimination and thereby protect them from even lower life satisfaction, we conducted an additional multi-level mediation analysis calculating both the indirect effect of structural stigma on life-satisfaction as mediated through concealment, and through the structural stigma → concealment → discrimination → life-satisfaction mediation path. Finally, to isolate the associations in our models to structural stigma operationalized as laws and policies only (and not also population attitudes), we re-ran this final analysis using an index of structural stigma composed of only country-level laws and policies. This permitted us to examine the stability of our associations across two different operationalizations of structural stigma.

## Results

### Descriptive statistics

The socio-demographic characteristics of the 6771 transgender people recruited to the EU-LGBT survey are presented in Table 1. The largest number of transgender participants lived in Germany (*n* = 1329) and the lowest number in Malta (*n* = 18). The large majority of respondents lived in an urban area and almost half (45.9%) were younger than 30 years old.

**Table 1:** Sociodemographic characteristics of individuals self-identifying as transgender in the European Union LGBT Survey 2012 (*n* = 6707)

	*n* (%)
Sex assigned at birth	
Female	2556 (38.1%)
Male	4151 (61.9%)
Age	
18–29 years	3073 (45.8%)
30–39 years	1536 (22.9%)
40–49 years	1164 (17.4%)
50–59 years	671 (10.0%)
60 years or older	263 (3.9%)
Ethnic minority status	466 (6.9%)
Level of education	
Less than university	3610 (53.8%)
University education	3097 (46.2%)
Household income	
Under the lowest quartile	2557 (38.1%)
Between the lowest quartile and median	1696 (25.3%)
Between the median and highest quartile	1320 (19.7%)
Above the highest quartile	1134 (16.9%)
Urbanicity	
Living in an urban area	5792 (86.4%)
Living in a rural area	915 (13.6%)
Relationship status	
Single	3206 (47.8%)
In a relationship, not living with a partner	1558 (23.2%)
Live with a partner	1943 (29.0%)
Sexual orientation	
Lesbian	1236 (18.4%)
Gay	1720 (25.6%)
Bisexual	1818 (27.1%)
Heterosexual	898 (13.4%)
Other	746 (11.1%)
Don’t know	289 (4.3%)

### Association between country-level structural stigma and life satisfaction, concealment of transgender identity, and day-to-day discrimination

**Fig. 2:**
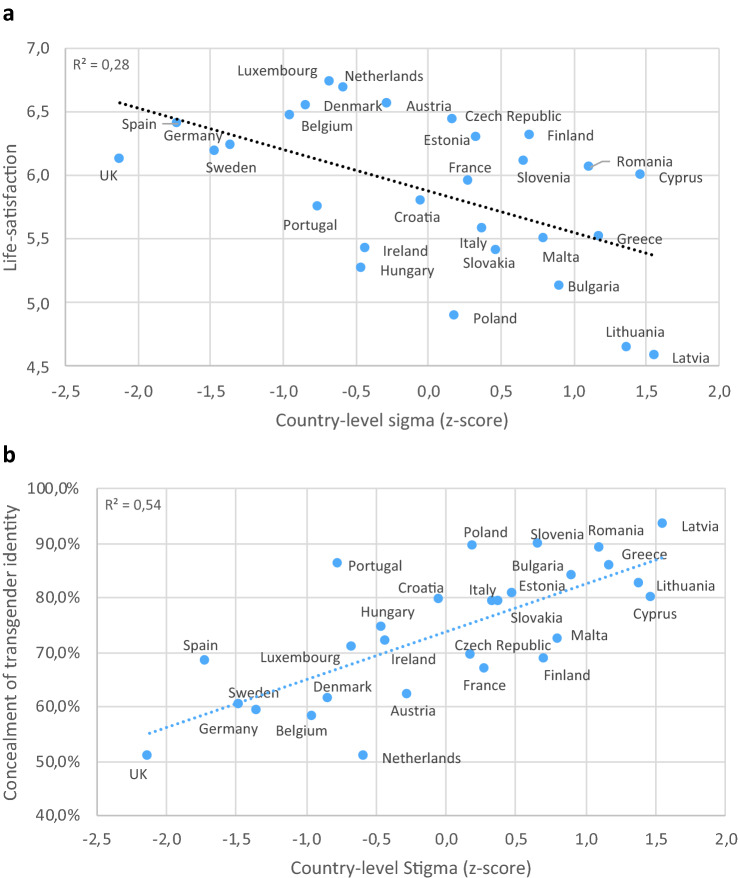
**a**, **b** Mean country-level self-reported life-satisfaction (**a**) and mean proportion of transgender people reporting high level of concealment of their transgender identity (**b**) among transgender people across Europe by country-level structural stigma

Multilevel regression models adjusted for age, ethnic minority status, education level, income, relationship status, and urbanicity (level 1) and for country-level Gini coefficient and life-satisfaction (level 2) showed significant associations between structural stigma and life satisfaction (adjusted *β* = − 0.188; 95% confidence interval [CI] − 0.364, − 0.013; *p* = 0.036; Fig. 2a), as well as between structural stigma and concealment of transgender identity (adj. *β* = 0.347; 95% CI 0.223, 0.471; *p* < 0.001; Fig. 2b). We found no significant association between structural stigma and day-to-day discrimination (adj. *β* = − 0.197; 95% CI − 0.482, 0.088; *p* = 0.176).

### Concealment as mediator of the association between country-level structural stigma and life satisfaction

The first multilevel mediation analysis examined the extent to which the relationship between structural stigma (level 2) and life satisfaction (level 1) could be explained by concealment of transgender identity (level 1). The model was adjusted for age, ethnic minority status, education level, income, relationship status, and urbanicity (level 1) and for country-level Gini coefficient and life-satisfaction at (level 2). The indirect effect of the association between structural stigma and life-satisfaction through concealment of transgender identity was significant (adj. *β* = − 0.336; 95% CI − 0.473, − 0.199; *p* < 0.001). When controlling for concealment of transgender identity, the relationship between structural stigma and life-satisfaction was no longer significant.

### Concealment of transgender identity as protection against day-to-day discrimination

**Fig. 3:**
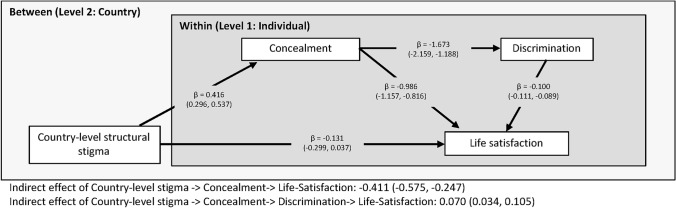
Multilevel mediation model of the association of country level structural stigma with life-satisfaction among transgender people across Europe mediated through transgender identity concealment and experiences of day-to-day discrimination

In a second multilevel mediation model, we investigated whether structural stigma predicts greater concealment to predict lower discrimination to predict life satisfaction. The results presented in Fig. 3 show that concealment significantly mediated the association between structural stigma and life satisfaction and that this association was still significant in the context of discrimination (adj. *β* = − 0.411; 95% CI − 0.575, − 0.247; *p* < 0.001). The indirect serial mediating effect of the association between country-level stigma and life-satisfaction (i.e., stigma → concealment → discrimination → life-satisfaction) was also significant (adj. *β* = 0.070; 95% CI 0.034, 0.105; *p* < 0.001). Therefore, the results indicate that concealment of transgender identity mediates the association between structural stigma and transgender people’s life satisfaction both directly and indirectly by reducing exposure to day-to-day discrimination.

To test the stability of our findings regarding associations with stigma at the structural-level, we re-ran this analysis using only country-level laws and policies as an indicator of county-level structural stigma and found a similar pattern of results (see Supplementary Fig. S1).

## Discussion

Although numerous non-probability studies indicate that transgender people experience more mental health problems, physical health concerns, and other indicators of poor life satisfaction than cis-gender individuals [1, 2, 6], to our knowledge, this is the first study to demonstrate an association between structural stigma and transgender people’s life satisfaction using an index of transgender-specific structural stigma. Using a unique dataset with responses from a large group of transgender people across Europe, this study documents potential mechanisms through which country-level structural stigma might exert its effect. In addition to showing that transgender people living in a country with a higher degree of structural stigma, measured by discriminatory laws, policies, and population attitudes, report lower life satisfaction compared to transgender people living in countries with lower degrees of structural stigma, results also show that this association could largely be explained by transgender people’s concealment of their identity. At the same time, findings indicate a potentially protective effect of identity concealment on transgender people’s exposure to day-to-day discrimination that further explains the association between structural stigma and life satisfaction.

These results are consistent with existing research demonstrating the negative impact of stigma and minority stress on sexual minority populations [9, 27]. Yet, this is the first know study to extend these findings to document the association between transgender-specific structural-level stigma and transgender people’s well-being. A particular strength of the study was its ability to examine manifestations of stigma across multiple socioecological levels, including structural (country-level discriminatory legislation and population attitudes), interpersonal (day-to-day discrimination), and individual (identity concealment), and their associations with our outcome, thereby extending the field of stigma studies that has tended to examine these levels of stigma in isolation [9].

Contrary to expectations and prior results among *sexual* minorities across Europe [27], we did not find a significant differences between high-stigma and low-stigma countries in terms of the degree to which transgender people were exposed to discrimination. One possible reason for this may be the concealability of one’s transgender status. Transgender people whose transgender identity is visible to others are exposed to more negative treatment than those whose identities are more concealable [6]. Supporting this, the results of this study show that transgender people who were open with their identity were subjected to more discrimination than transgender people who concealed their identity and that concealment was more common in countries with high structural stigma toward transgender persons. These findings suggest that transgender people perhaps make the tradeoff between the protections and constraints of concealment at least partially informed by their social context, consistent with decision-making models of identity disclosure [28, 29]. At the same time, numerous studies have demonstrated the negative impact of identity concealment on well-being [15, 27]. While transparency about one’s identification can facilitate access to support and belongingness from similar others [30] and appropriate and affirmative healthcare [3], this study indicates that openness is associated with higher exposure to day-to-day discrimination and lower life satisfaction. Therefore, the safety of one’s structural and interpersonal surroundings are paramount in considerations of identity disclosure or visibility and clinicians should take all necessary steps to ensure and support such safety.

This study emphasizes the importance of reducing stigma across structural, interpersonal, and individual levels. Specifically, findings suggest that implementing changes in legislation and population attitudes toward transgender people can perhaps yield a significant impact in life satisfaction. Of particular importance for transgender people are efforts to reduce stigma in healthcare settings since many transgender people rely on such services. Examples of changes in legislation and policies that could reduce stigma in healthcare settings are removal of gender identity disorder diagnoses, compulsory requirements of sterilization to access gender affirming care, and other barriers to change of legal gender. Even though efforts to reduce discriminatory legislation and policies have been made in several European countries during the past several years, day-to-day discrimination is commonly reported among transgender people in all European countries, regardless of structural stigma, suggesting that bias-reduction interventions might also be an important route to improving transgender people’s wellbeing [31]. Finally, supporting personal resilience through mental health interventions could help transgender people cope with the stress of structural and interpersonal forms of stigma, in ways that support personal and community empowerment in the face of these systemic conditions [32].

Despite being derived from a large international dataset of transgender people and taking advantage of multi-level assessments of transgender stigma, these study results must be considered in light of several limitations. First, participants were self-selected into the study and it is unknown how representative participants might be of the total transgender population in their respective countries. Second, we had limited information about transgender-specific experiences and gender identities of participants, thereby making it impossible to examine other mechanisms or stratify analyses based on important within-group characteristics. Without having assessed stigma directed toward other marginalized social statuses across levels, such as ethnic/racial minority, immigrant, or socioeconomic, the present study could not permit examining intersections of this stigma with stigma directed to gender minority identity. Research using an intersectional framework represents an important future direction for examining potential moderators of the associations found here [33]. Third, this study relies on a single-item indicator of life satisfaction. The item used, however, has shown high consistency across large-scale studies in many different country contexts and across time [17, 20], and it has shown a strong link with a multitude of indicators of mental, physical, and social health [16, 17]. Nevertheless, only future research assessing broader aspects of health and wellbeing will be able to further increase our knowledge about in impact of stigmatizing environments on transgender people. Fourth, the questions used to assess day-to-day discrimination and concealment of transgender status have not been extensively validated. It is therefore unclear how accurately these non-validated measures might have captured these experiences of study participants. Future measures validation studies among transgender people is needed to strengthen our understanding of the impact of these factors on transgender people’s life satisfaction. Fifth, the cross-sectional nature of the study design makes it impossible to draw causal conclusions and only future studies with longitudinal designs can help clarify the causal pattern though which changes in stigmatizing environment might causally influence the lives of transgender people. Finally, since the study was conducted among European Union member states it is difficult to assess how well these findings represent the situation of transgender people outside of Europe. Several of the European countries included in our study are among the least stigmatizing towards transgender people both regarding legislation/policies and population attitudes, it is possible that the pattern of associations among stigma, discrimination, and identity concealment look somewhat different in countries with even higher levels of stigma than those represented within the European context. Population-representative studies outside of Europe can test the generalizability of the present findings.

In sum, this study finds a negative association between structural stigma and life satisfaction among transgender people in Europe. The differences in transgender people’s life satisfaction across countries can largely be explained by how open or concealed they are about their transgender identity. The results show the strong role that structural forms of stigmatization can play in transgender people's lives and wellbeing. The results emphasize the importance of changing discriminatory legislation and negative population attitudes to improve the situation of transgender people, and also highlight targets for intervention at the interpersonal and individual levels.

## Supplementary Information

Below is the link to the electronic supplementary material.

Supplementary file1 (DOCX 39 KB)

## Data Availability

Data is available under special license agreement at the UK Data Service (https://ukdataservice.ac.uk/).

## Code availability

Please, contact the corresponding author.
