# Supplementary material for: Country-level structural stigma, identity concealment, and day-to-day discrimination as determinants of transgender people’s life satisfaction
Source: Soc Psychiatry Psychiatr Epidemiol. 2021 Feb 13;56(9):1537–45. doi: 10.1007/s00127-021-02036-6 (PMC8429389; doi:10.1007/s00127-021-02036-6)
Supplement: Supplementary file 1 — Supplementary file1 (DOCX 39 KB) [file 127_2021_2036_MOESM1_ESM.docx]

**Figure S1** Multilevel mediation model of the association of country level structural stigma (measured with an index of discriminatory legislation and policies, removing population attitudes) with life-satisfaction among transgender people across Europe mediated through transgender identity concealment and experiences of day-to-day discrimination

**
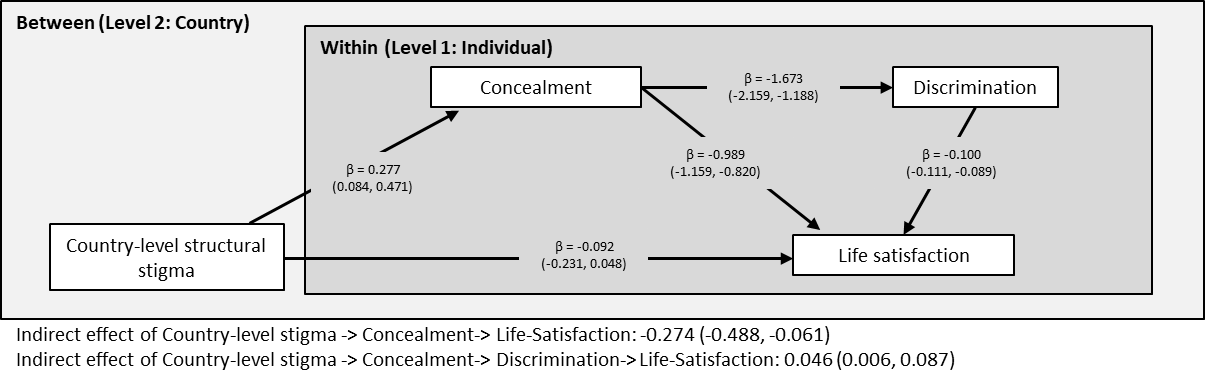
**
